# An environmental scan of Ontario Health Teams: a descriptive study

**DOI:** 10.1186/s12913-023-09102-6

**Published:** 2023-03-08

**Authors:** Claire Sethuram, Tess McCutcheon, Clare Liddy

**Affiliations:** 1 C.T. Lamont Primary Health Care Research Centre, Bruyère Research Institute, Ottawa, ON K1R 6M1 Canada; 2 Department of Family Medicine, University of Ottawa, Ottawa, ON K1G 5Z3 Canada; 3 Ontario eConsult Centre of Excellence, The Ottawa Hospital, Ottawa, ON K1H 7W9 Canada

**Keywords:** Primary health care, Health services, Health equity, Integrated care, Transitions of care, Ontario health teams

## Abstract

**Background:**

Ontario Health Teams (OHTs) are an integrated care system introduced in Ontario, Canada in 2019 after the 14 Local Health Integrated Networks (LHINs) were dissolved. The objective of this study is to give an overview of the current state of the OHT model’s implementation, and what priority populations and transitions of care models were identified by OHTs.

**Methods:**

This scan involved a structured search for each approved OHT of publicly available resources with three main sources: the full application submitted by the OHT, the OHT website, and a Google search with the name of the OHT.

**Results:**

As of July 23, 2021, there were 42 approved OHTs and nine transitions of care programs were identified across nine OHTs. Of the approved OHTs, 38 had identified ten distinct priority populations, and 34 reported partnerships with organizations.

**Conclusions:**

While the approved OHTs currently cover 86% of Ontario’s population, not all OHTs are at the same stage of activity. Several areas for improvement were identified, including public engagement, reporting, and accountability. Moreover, OHTs’ progress and outcomes should be measured in a standardized manner. These findings may be of interest to healthcare policy or decision-makers looking to implement similar integrated care systems and improve healthcare delivery in their jurisdictions.

**Supplementary Information:**

The online version contains supplementary material available at 10.1186/s12913-023-09102-6.

## Background

In 2019, the Ontario government passed the *Connecting Care Act, 2019* that initiated a redesign of the existing health system. Considered “one of the largest reforms to the provincial health system” by the Ontario Medical Association [1] (np)], the Act includes dissolving its 14 Local Health Integrated Networks (LHINs) and replacing them with small, local teams within communities, called Ontario Health Teams (OHTs), spread across the province [2, 3]. Similar to other areas of Canada, the country’s most populous province, Ontario [4], was struggling to deliver healthcare that is continuous and accessible to its residents. Whereas the LHINs were regionally based and autonomously run, OHTs are community based and managed by a newly created agency, Ontario Health, with the vision that by transferring the bureaucratic load to a central agency and narrowing their scope, OHTs will be more patient-centered than LHINs and the system more cost effective [5, 6]. Eliminating the silos that exist between the various internal organizations and integrating services would not only create safer and more financially efficient care delivery, but also better patient experiences and healthier communities [2]. Such models of care, where healthcare providers work as a team, rather than separate entities, have already been identified and implemented as a directional goal in healthcare delivery. For example, the College of Family Physicians of Canada’s patient’s medical neighbourhood (PMN) model [7] aims to provide safe, comprehensive, and coordinated quality care that is integrated across an entire care network while remaining patient-centered and rendering services accessible to a specific population [8, 9].

In the case of OHTs, the intent was to expand beyond primary care and encourage groups of healthcare providers, such as physicians, home and community care organizations, and hospitals, defined by existing patterns of access and referral, to formally link within the healthcare system [10–12]. The aim is to design a health system tailored to a defined population and allow patients to access healthcare and transition from provider to provider seamlessly within their community [10]. Aligned with the PMN’s goals of fostering a collaborative environment between healthcare professionals by integrating virtual care, necessary infrastructure, and coordination of care, groups of providers were invited to form a team and submit a proposal describing their plans for transforming care, implementation, and creating community partnerships. In this proposal, teams were also asked to identify a priority population, such as seniors, people living in rural areas, or people living with dementia, to focus care redesign and improvement efforts during the initial implementation of the OHT over its first year [13]. This report aims to give an overview of the current state of the OHT model’s implementation. We were specifically interested in seeing what priority populations and transitions of care models were identified by OHTs, as this was an area of interest identified by our one of our funders, Innovations Strengthening Primary Health Care Through Research (INSPIRE-PHC).

## Methods

### Setting

**Fig. 1:**
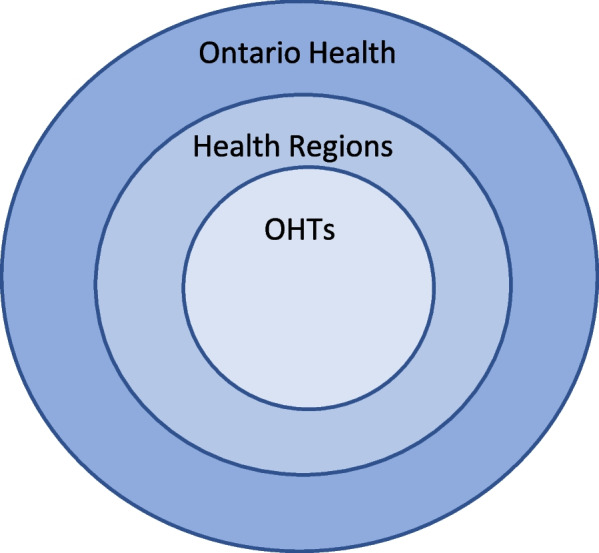
Relationship between Ontario Health, health regions, and OHTs

The study was conducted in Ontario, Canada. In Canada, public healthcare is managed at the provincial level. When the LHINs came into effect in 2007, Ontario was divided into 14 geographic health regions. These regions stood until the *Connecting Care Act* was enacted in 2019, creating Ontario Health, which has divided the province into health regions that cover larger areas than the LHINs. At the time of data collection (Summer 2021) there were five health regions: West, Central, Toronto Central, East, and North. Since that time, the North region has split into North West and North East. Ontario Health utilizes the regions to “work with local community and health care partners”, track and evaluate health system performance, and oversee OHTs [14] (np)]. Because Ontario covers approximately 1.1 million km^2^ with approximately 14.9 million residents [4] unequally distributed across the province, each region has unique needs and challenges in delivering healthcare to its residents. Each region contains multiple OHTs that are healthcare partnerships at the community level that are funded by and report to Ontario Health (Fig. 1).

### Study design and approach

Our team conducted this environmental scan between June and August 2021 based on the process outlined by the Canadian Agency for Drugs and Technologies in Health [15]. First, we found a list of approved OHTs on the Ontario Ministry of Health and Ministry of Long-Term Care webpage [16]. For each approved OHT, we used a structured search of publicly available resources with three main sources:

- 1. The full application submitted by the OHT, to identify the initial plans of the OHT, including Year 1 target populations, attributed population size, and partners;
- 2. The OHT website (if available), to identify any updates on work related to Year 1 targets;
- 3. A Google search with the name of the OHT (e.g., “Burlington OHT”), to identify any updates related to Year 1 targets that may not have been provided on the OHT website. We limited this search to the first five pages of results.

### Inclusion/exclusion criteria for google search

We included the resources that described updates on the OHT’s activities (e.g., programs related to their priority population for Year 1), and those that were publicly available and published after the full application was approved.

We excluded resources regarding COVID-19 activities that were not initially outlined in the full application, such as information about testing sites and vaccine clinics. This exclusion criterion allowed us to focus on the OHT’s implementation of the plans outlined in the full application. While we realize that the pandemic was of great significance, it is also important to ensure that the OHTs are not pausing the work they set out to accomplish, such as increasing resources for mental health and addictions or improving transitions care of the elderly, prior to the declaration of the pandemic [17, 18]. In fact, integrated care with strong partnerships is even more significant during this time [19].

### Data extraction and description

For each approved OHT, we recorded the following information from their full applications in an Excel spreadsheet: name of OHT, date of approval, population(s) of focus, population size, number of affiliated organizations or partners, website, and additional notes. After data extraction, we summarized and tabulated the findings from the full applications, OHT websites, and Google searches of all approved OHTs. Any findings related to transitions of care were also summarized together. We used summary statistics and frequencies to describe commonalities across approved OHTs, including Ontario Health region, priority populations, partnerships, and population size.

### Ethics

Since we collected all materials for data analysis from publicly available resources on the internet, no ethics approval was required, as per the policy of our local research ethics board, the Ottawa Health Sciences Network.

## Results

As of July 23, 2021, the Government of Ontario had approved forty-two OHTs [11]. Twenty-four OHTs were approved before the COVID-19 pandemic was declared in March 2020. There was a seven-month gap between the first and second OHT approval cohorts. Eighteen OHTs were approved after the onset of the pandemic. These approved OHTs currently cover 86% of Ontario’s population [11].

Of the approved OHTs, 38 OHTs had identified ten distinct priority populations. Most OHTs (66%; *n* = 25) had listed more than one priority population. The top three populations identified were Mental Health and Addiction (66%; *n* = 25), Seniors (61%; *n* = 23), and Palliative Care (29%; *n* = 11). The remaining priority populations, along with the number of OHTs that identified these groups as a priority for their region, are listed in Table 1.

**Table 1:** OHT-identified priority populations (*n* = 38)

Priority population Identified by OHT	Number of OHTs	Percentage
Mental Health and Addictions	25	66%
Seniors	23	61%
Palliative	11	29%
Chronic conditions	4	11%
Homelessness and Precarious Housing	4	11%
Dementia	2	5.3%
Rural	2	5.3%
Acute GI/GU	1	2.6%
High Health System Users	1	2.6%
Refugee	1	2.6%

All five health regions had approved OHTs. Of the five Ontario Health Regions, the Central Region had the most approved OHTs, with 17 (40%). The West Region had the second most with 12 approved OHTs (29%), surpassing the East and North Regions, with only four approved OHTs each. Of the 42 approved OHTs, the majority (*n* = 32) reported their population size in their initial applications and had an average population of 325,902; the Ottawa OHT’s application reported the largest population coverage at 934,242. In comparison, Muskoka and Area OHT reported the smallest population coverage at 64,445 people.

Thirty-four OHTs (80%) provided information on the number and types of partnerships they have developed, including organizations, patient or clinician partners, and community or government agencies. Of the OHTs that had self-reported their number of partnerships, 13 (38%) had 1–20 partners, 12 OHTs (35%) reported 21–40 partners, six OHTs (18%) reported 41–60 partners, one OHT (3%) reported 61–80 partners, and two OHTs (6%) had more than 80 partners. Specific numbers of patient partners were not available. We found limited information on the patient and community engagement initiatives across OHTs and could not find a website for eight of the OHTs (19%).

### Transitions of care programs

We identified transitions of care models during the environmental scan (Table 2). Many of these programs were identified in OHTs’ full applications as Year 1 projects, and they have been implemented in various OHTs to improve transitions of care. However, we found no evidence regarding their utilization, impact, and effectiveness. Brief descriptions of these innovative approaches to transitions of care can be found in Appendix A.

**Table 2:** Transitions of care models

Transitions of care resource	Ontario Health Team(s)	Ontario Health Region
Community paramedicine program	Chatham-Kent	West
Surgical transitions virtual care	Hamilton Health Team	West
Three-page form improving communication during transfers	Eastern York Region and North Durham	Central
High-Intensity Supports at Home (HISH) Program	Connected Care Halton, Eastern York Region and North Durham	Central
Seniors Home Support (SHS) Program	Eastern York Region and North Durham	Central
North York Community Access to Resources Enabling Support (North York CARES)	North York Toronto Health Partners	Central
Southlake@home and COVID@home programs	Southlake Community	Central
COVID-19 hospital-to-home transitions	Hills of Headwaters	Central
NP-led clinic focused on transitions for children	Kids Come First	East

## Discussion

### Summary

This descriptive analysis of OHTs provides an overview of the status, transitions of care models, and reporting from each OHT approved before September 2021. Of the approved OHTs, 38 had identified ten distinct priority populations, and 34 reported partnerships with local organizations. We also identified nine transitions of care programs across nine OHTs. The approved OHTs currently cover 86% of Ontario’s population; however, not all OHTs are at the same stage of activity. Based on these findings, we have identified several areas for improvement, including public reporting, patient and public engagement, and accountability for meeting deliverables.

### Reporting

Initially when designing the study, we were interested in learning about the OHTs’ active projects from their public reporting but as we begun the data extraction process, we soon realized there was an absence of publicly available information. While there is a standardized process to approve OHTs, at the time of this study there was no standard for reporting outcome measures and evaluations. In November 2022, the Ontario Ministry of Health released new requirements for OHTs that included a section entitled “Cultivating Consistency in OHT-Led Public Communications” that lays out expectations and standards for how communications are delivered to the public [12, 20]. During the application process for approval, there are key steps and stages that must be followed and reporting requirements at certain time intervals. Additionally, there is clear maturity from the “in discovery” phase to “in development,” followed by “OHT candidate” stage and finally, “approved OHT” [21]. Moreover, all OHT candidates must submit the standardized OHT application outlining their plans and make it publicly available. However, once the OHT has been approved, there is no standard method of reporting to the public. This is of particular concern since a recent report by the National Academies of Sciences, Engineering, and Medicine on the realization of high-quality primary and community care asserted that processes for keeping parties accountable to meeting their goals were paramount to the success of a strong health system [22]. The lack of information relating to the evaluation and accountability of each OHT is notable as these data are essential to facilitate effective quality improvement and research at a systems-level; however, there is evidence of improvement here in the coming years in the Ontario organization, Health System Performance Network (HSPN). HSPN is conducting on-going OHT evaluations that encompass both the development of OHTs following approval and health and system improvement indicators of OHT attributable and priority populations [23].

### Population coverage at full approval

Currently, 42 OHTs are approved and cover 86% of Ontario’s population. Once the OHTs in development reach approval, 99% of Ontario’s population will be covered; however, it should be noted that the OHTs currently in development are not all at the same stage in the approval process and do not have the same level of activity [11].

One pitfall of innovation in this sector is that the model might not provide adequate or equitable coverage. For example, the Family Health Team (FHT) model has been cited as a solution to increase access to and quality of primary care [24, 25]. However, a recent study in Ontario found that the patient rosters of FHTs tend to be wealthier, healthier, located in rural regions and have low immigrant representation [26]. Once all OHTs are implemented, the model will offer greater population coverage relative to FHTs, which were only partially implemented in Ontario. To ensure equitable population coverage is offered, it must be measured and compared between teams. We recommend the development of a maturity index after OHT approval to monitor how each OHT progresses that includes the adoption of standardized equity outcome measures.

### Patient medical neighbourhood

OHTs are moving towards the PMN model by creating integrated care to serve communities based on their needs, supported by local partners and organizations with adequate financial and administrative resources. Although there is a high level of partner engagement with an OHT’s community, OHTs provide little information to engage physicians [27]. This is consistent with findings from other studies on OHT implementation. For example, in a qualitative study by Embuldeniya et al. (2020), physicians reported being unaware of how OHT implementation would affect them. A report by the National Academies of Sciences, Engineering, and Medicine highlighted the importance of physician and community engagement as an essential component of strong primary and community care [22]. Hence, there is a need for OHTs to strengthen public engagement and communication with those in the community to ensure that health organizations are included in the process. Some OHT websites had not been updated since their approval in 2019 and did not outline any information for community members looking to get involved. This indicates that patient engagement, community co-design, and patient-reported outcomes need to be further addressed in the OHT model.

### COVID-19

There are notable differences between the OHTs formed before and after the beginning of the COVID-19 pandemic in March 2020. Overall, it was noted that some OHTs approved before the pandemic were not as successful in meeting their targets for Year 1 priority populations. Instead, it seems as though they had to pivot very quickly to focus on demands associated with COVID-19 and were unable to continue with their planned work outlined in the application. In comparison, OHTs approved after the onset of the pandemic often had COVID-19 strategies built directly into their full applications. As a result, these groups moved forward with both their COVID-19 work and their Year 1 priority population focus.

### Strengths and limitations

To the best of our knowledge, our study is the first to examine the current status of each approved OHT since the initial 24 OHTs were approved and the onset of COVID-19 [28]. It offers a succinct description of progress made before August 2021 and identifies gaps that should be improved moving forward. Limitations of this study include its short timeframe and exclusion of OHTs approved after August 2021. Furthermore, this study relies on data derived from publicly available resources only; as a result, important information that is not reported to the public might not be included in this analysis. We also excluded articles that focused on COVID-19 activities that were not initially outlined in the full application. Regardless, this study provides an overview of the current state of OHT implementation and provides recommendations to enhance public engagement and to measure OHT maturity.

## Conclusion

This paper aimed to describe the current state of OHTs across the province. Currently, 42 OHTs have been approved, covering 86% of the province’s population; in Ontario, this accounts for almost two million residents without coverage by an OHT. Once the OHTs in development are approved, 99% of the population will be covered. Concerns, however, remain standards of reporting, accountability, and equitable coverage across the OHTs. This reflects one of the biggest challenges in implementing healthcare systems: ensuring sufficient population coverage and equitable access to care for all. It is recommended that more effort be made to measure OHTs’ progress and outcomes in a standardized manner.

## Supplementary Information

**Additional file 1.**

## Data Availability

All data used in this project was collected from http://www.google.ca. Questions about data, its collection, and the analysis can be directed to the corresponding author, Dr. Clare Liddy, at cliddy@uottawa.ca.
