# Supplementary material for: An environmental scan of Ontario Health Teams: a descriptive study
Source: BMC Health Serv Res. 2023 Mar 8;23:225. doi: 10.1186/s12913-023-09102-6 (PMC9993364; doi:10.1186/s12913-023-09102-6)
Supplement: Supplementary file 1 — Additional file 1. [file 12913_2023_9102_MOESM1_ESM.docx]

**Appendix A:** Additional information about each transitions of care program

**Improving communication during transfers:** three-page form designed to improve communication during transfers of patients from long-term care or retirement homes to the emergency department and vice versa.

**Community paramedicine program:** to improve transitions from one provider to another by providing tailored support for complex patients. Paramedics can also provide in-home visits to patients as they transition from acute care settings back to community

**High-intensity supports at home (HISH) program:** provides support for up to 35 high risk seniors in the community who are on the waitlist for long-term care placement in the Halton region. Similar program exists in EYRND OHT.

**Seniors Home Support (SHS) Program:** An integrated primary care program delivered by an interdisciplinary team to home-bound seniors who for medical, social or cognitive reasons cannot access office-based primary care services. The SHS Program is delivered by a care team which includes primary care physicians, a nurse practitioner, an occupational therapist, a pharmacist, a social worker and a chiropodist. Available to: seniors and frail elderly, including those who are isolated, marginalized people in quarantine and self-isolation including adults and families, newly discharged patients and clients at risk of returning to hospital, older adults who become ill while in the community and informal caregivers overwhelmed by additional challenges of caring for loved ones while sheltering at home.

**North York CARES:** Provides care and support for patients, who would otherwise be in hospital, so they can safely stay at home while waiting for a bed in long-term care or another setting. The program also benefits the hospital by creating more available beds for those who need them most.

**Southlake@home:** Designed to improve care for older adults with complex medical and social needs who are transitioning home after an admission to Southlake, requiring significant support to stay well at home.

**COVID@home:** Enables COVID-19 positive patients who are experiencing moderate symptoms (e.g. positive chest x-ray for pneumonia and normal O2 saturation) to be monitored at home by a remote clinical team for optimal clinical outcomes. Monitored twice daily with smart devices, collaborative program involving PCPs, assessment centre staff, ED on-call physician, Southlake Telehomecare Nurses. If condition escalates, alert is activated.

**COVID-19 transitions:** During the pandemic, the Hills of Headwaters OHT moved quickly to set up a virtual process between the local hospital and the Dufferin Area FHT. When hospitalized COVID-19 patients are stabilized and discharged – but require follow-up care – a respiratory therapist at the FHT remotely monitors these patients. Paramedics are also on board as first responders in a patient emergency.

**NP-led clinic:** new clinic led by a nurse practitioner. The NP-led clinic provides quick access to health services for children transitioning in or out of the care of the Children’s Aid Society of Ottawa (CASO), often because they don’t have a primary care provider in the community. The NP also oversees a meeting to ensure each child and family gets the support they need.
